# Supplementary material for: T-cell dysfunction in the glioblastoma microenvironment is mediated by myeloid cells releasing interleukin-10
Source: Nat Commun. 2022 Feb 17;13:925. doi: 10.1038/s41467-022-28523-1 (PMC8854421; doi:10.1038/s41467-022-28523-1)
Supplement: Supplementary file 1 — Supplementary Information [file 41467_2022_28523_MOESM1_ESM.pdf]

T-Cell Dysfunction in the Glioblastoma Microenvironment is Mediated by Myeloid Cells Releasing Interleukin-10

SUPPLEMENTARY INFORMATION

Vidhya M. Ravi[1,2,3,4,5,#], Nicolas Neidert[1,2,3,6,#], Paulina Will[1,2,3,#], Kevin Joseph[1,2,3], Julian P. Maier[1,2,3], Jan Kückelhaus[1,2,3], Lea Vollmer[1,2,3], Jonathan M Goeldner[1,2,3], Simon P. Behringer[1,2,3,6], Florian Scherer[3,7], Melanie Boerries[3,8,9,10], Marie Follo[3,7], Tobias Weiss[11], Daniel Delev[12,13], Julius Kernbach[12,13], Pamela Franco[2,3,6], Nils Schallner[3,14], Christine Dierks[3,7], Maria Stella Carro[2,3], Ulrich G. Hofmann[2,3,4], Christian Fung[2,3], Roman Sankowski[3],[15], Marco Prinz[3,15,16,17], Jürgen Beck[2,3,17], Henrike Salié[3,16,18], Bertram Bengsch[3,16,18], Oliver Schnell[2,3,6,#], Dieter Henrik Heiland[1,2,3,9,10,#,*]

[1]Microenvironment and Immunology Research Laboratory, Medical Center-University of Freiburg, Germany

[2]Department of Neurosurgery, Medical Center-University of Freiburg, Germany

[3]Faculty of Medicine, University of Freiburg, Germany

[4]Neuroelectronic Systems, Medical Center-University of Freiburg, Germany

[5]Freiburg Institute for Advanced Studies (FRIAS), University of Freiburg, Germany

[6]Translational NeuroOncology Research Group, Medical Center-University of Freiburg, Germany

[7]Department of Medicine I, Medical Center-University of Freiburg, Germany.

[8]Institute of Medical Bioinformatics and Systems Medicine, Medical Center-University of Freiburg, Germany.

[9]Comprehensive Cancer Center Freiburg (CCCF), Faculty of Medicine and Medical Center-University of Freiburg, Germany.

[10]German Cancer Consortium (DKTK), partner site Freiburg.

[11]Department of Neurology and Brain Tumor Center, University Hospital Zurich and University of Zurich, Switzerland

[12]Department of Neurosurgery, RWTH University of Aachen, Germany

[13]Neurosurgical Artificial Intelligence Laboratory Aachen (NAILA), Department of Neurosurgery, RWTH University of Aachen, Germany

[14]Department of Anesthesiology and Critical Care Medicine, Medical Center-University of Freiburg, Germany

[15]Institute of Neuropathology, Medical Center-University of Freiburg,

[16]Signalling Research Centre BIOSS and CIBSS, University of Freiburg, Germany

[17]Center for NeuroModulation (NeuroModul), University of Freiburg, Freiburg, Germany

[18]Department of Medicine II: Gastroenterology, Hepatology, Endocrinology, and Infectious Disease, Medical Center- University of Freiburg, Germany.

# 'These authors contributed equally

*** Corresponding author**

Corresponding author:
Dieter Henrik Heiland
Department of Neurosurgery
Medical Center University of Freiburg
Breisacher Straße 64
79106 Freiburg
-Germany-
Tel: +49 (0) 761 270 50010
Fax: +49 (0) 761 270 51020
E-mail: dieter.henrik.heiland@uniklinik-freiburg.de

Supplementary Figure 1

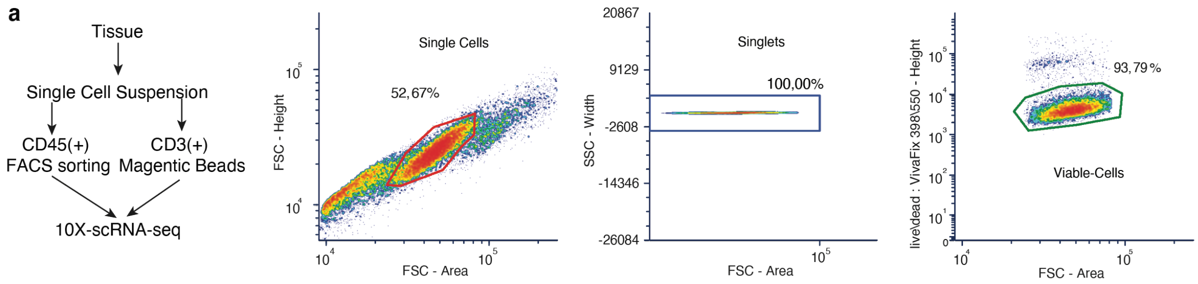

Supplementary Figure 1: a) Workflow and representative FACS images.

Supplementary Figure 2

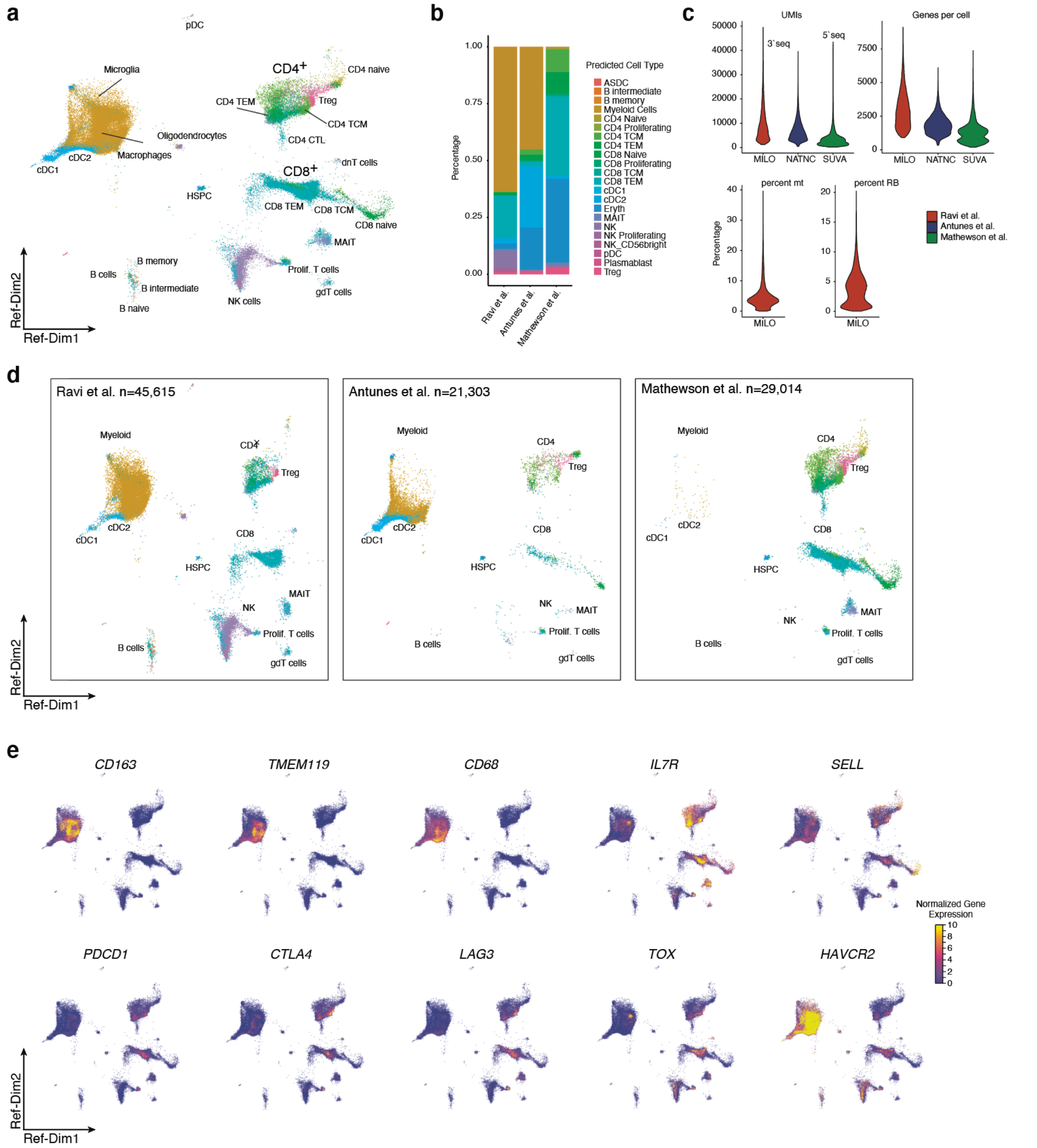

Supplementary Figure 2: a) Horizonal integration with WNN of all lymphoid/myeloid datasets. 2D representation of the reference dimensions indicate the different annotated cell types. b) Bar plot of cell types across datasets. c) Violin plots of data quality across datasets. d) Comparison of annotated cell types in individual datastes. e) Gene expression of marker genes in the full integrated dataset.

Supplementary Figure 3

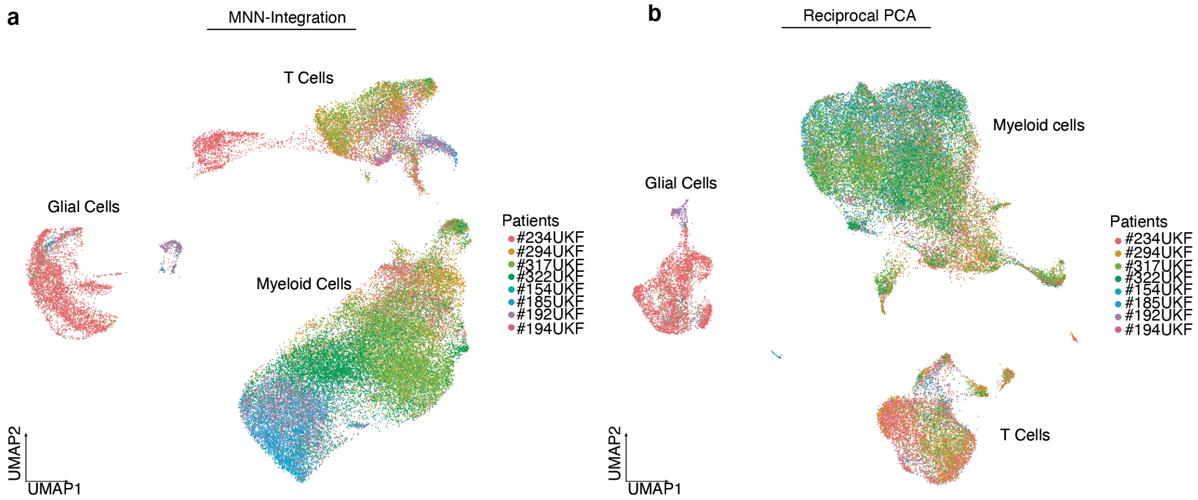

Supplementary Figure 3: a-b) Validation of the MNN (a) and rPCA(b) integration.

Supplementary Figure 4

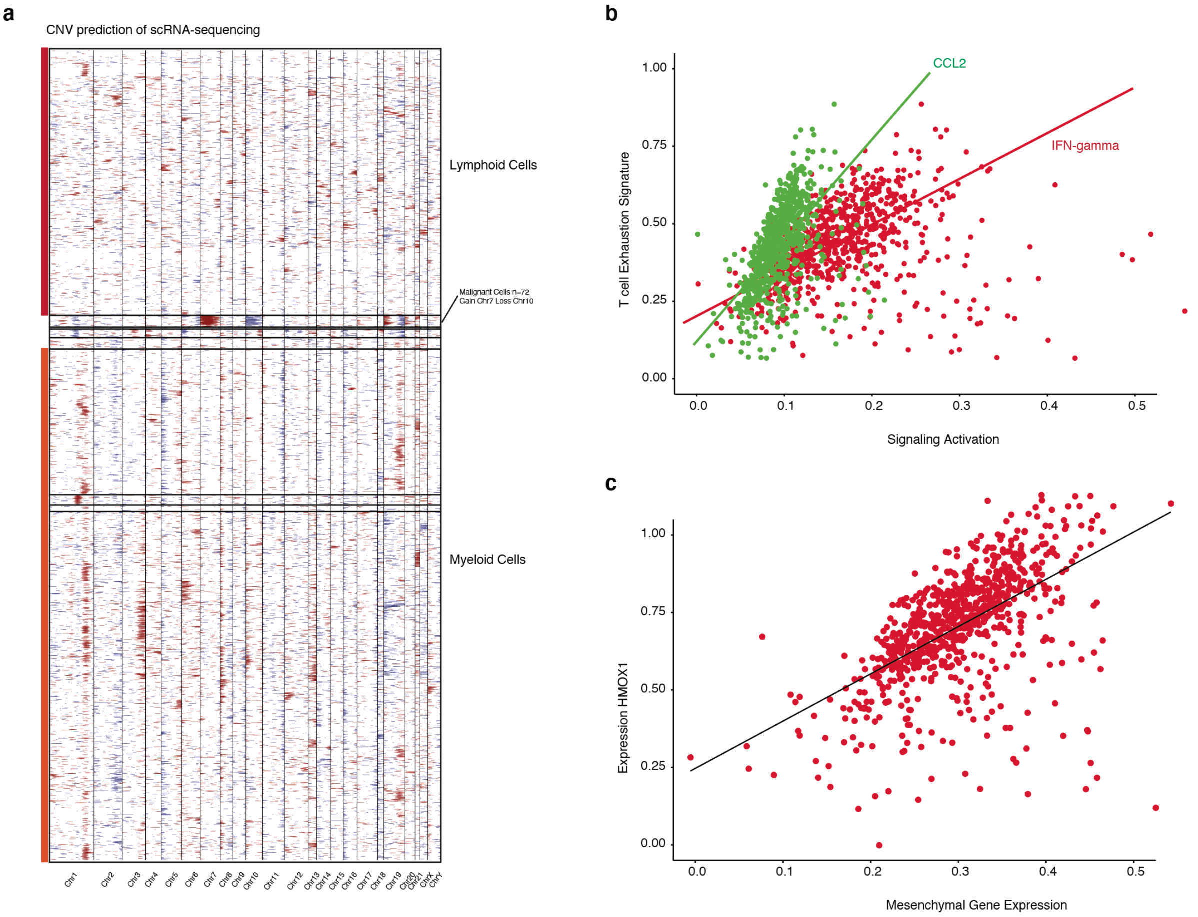

Supplementary Figure 4: a) Copy-number alterations based on single cell data. Only a small subset of tumor cells was found in the OPC cluster. b) Spatial correlation plots indicate the spatial overlap of T cell exhaustion (y-axis) and CCL2 (green, $R2=0.87$, padj.<0.05) and IFN-gamma (red $R2=0.63$, padj.<0.05) response. c) Spatial correlation plots indicate the spatial overlap of HMOX1 expression (y-axis) and the mesenchymal subgroup ($R2=0.72$, padj.<0.05)

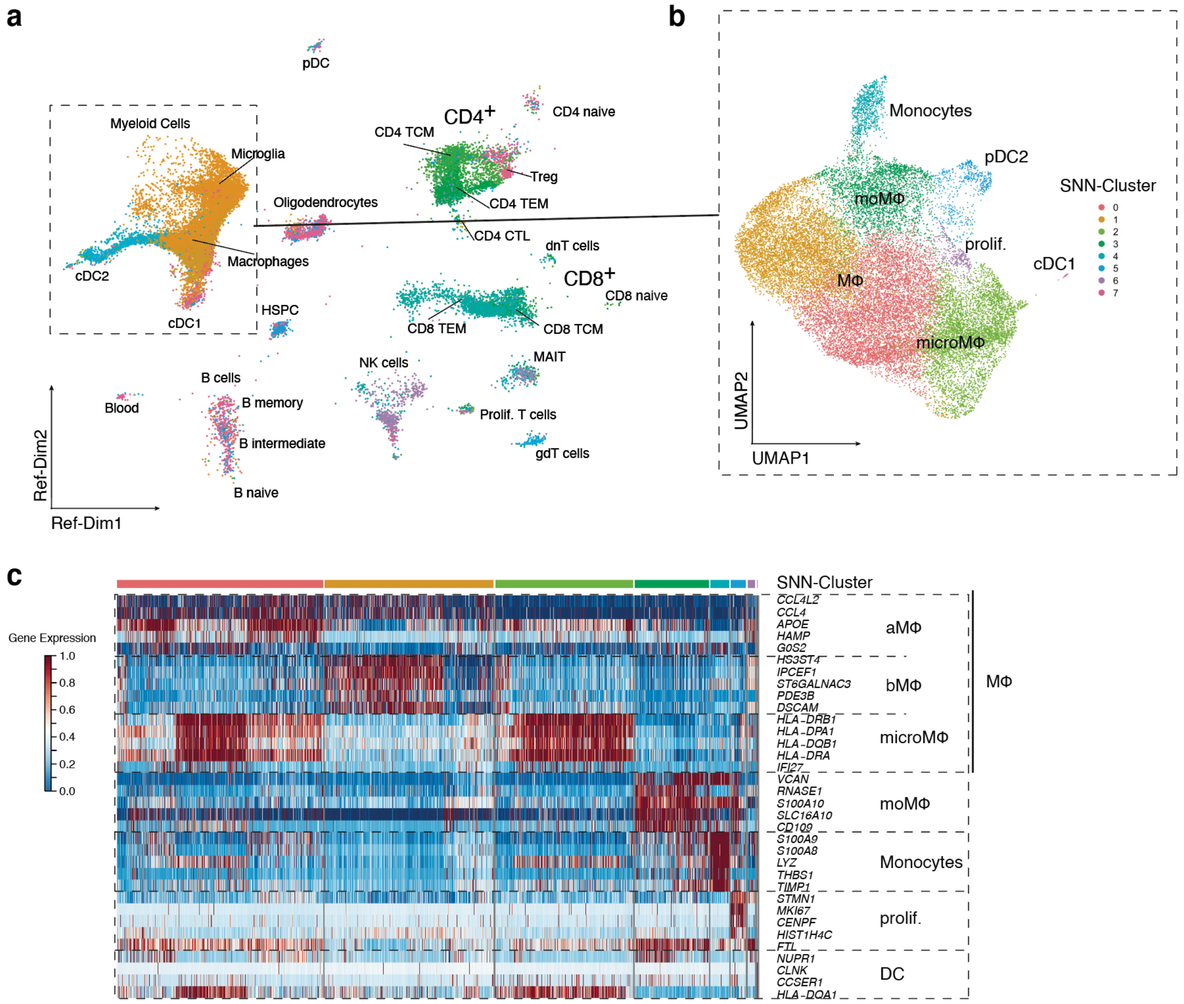

Supplementary Figure 5: a-b) Cluster of myeloid cells. The cells are isolated from the reference cell type annotation map and processed by MNN integration followed by SNN clustering. The number of clusters are determined by the optimal cluster stability using cluster-trees. c) Illustration of an expression heatmap of the top 5 marker genes of each cluster.

Supplementary Figure 6

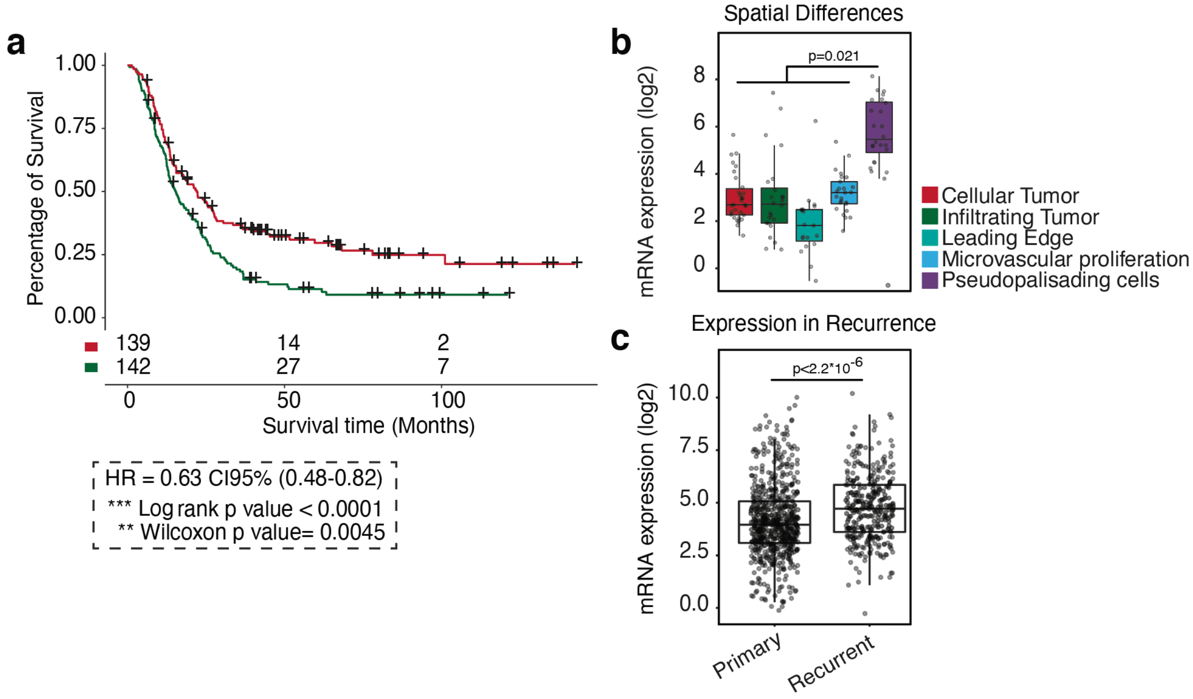

Supplementary Figure 6: a) Kaplan-Meier survival estimation of HMOX1 high/low expression GBM. b-c) Expression of HMOX1 in different regions of the tumor (b) and in de-novo and recurrent stage (c). P-values are determined by one-way ANOVA (b), one-sided T test (c) and log-rank statistics (a).

SUPPLEMENTARY Methods

1. Nearest Functional Connected Neighbors (NFCN)

1.1. Background of the NFCN development

Over the recent years, several algorithms have been developed to infer cellular connections based on receptor ligand expression profiles. These algorithms allow us to predict potential cellular interactions from single cell transcriptomic data, the most common being CellPhoneDB[1], NicheNet[2] and CellChat[3]. Both algorithms require previously annotated clusters, using the resulting cell populations to infer connectivity. These algorithms are highly recommended for *de-novo* exploration of data but lack accuracy when defined signaling pathways across cell populations are explored. In the context of our investigation, we aimed to develop an unsupervised algorithm that can predict the cellular interactions of a target and source population, without prior cluster annotations. Our novel NFCN algorithm (NFCN2) is tailored to predict cell pairs that are most likely to be connected. These predicted cellular interactions are further corrected using spatially resolved transcriptomics and **p**hysically **i**nteracting **c**ell-seq (PIC-seq).

NFCN2 can be seen as supplementary to CellPhoneDB and CellChat, where the inferred Cell-Cell interactions are further validated. In the current version, CellChat is implemented as the first step of the algorithm, which opens up the possibility to combine de-novo exploration and validation within the NFCN pipeline. To ensure compatibility with existing workflows, we use Seurat objects as a common S4-object format. The output from the NFCN workflow is stored as an “assay” in the S4 Seurat object.

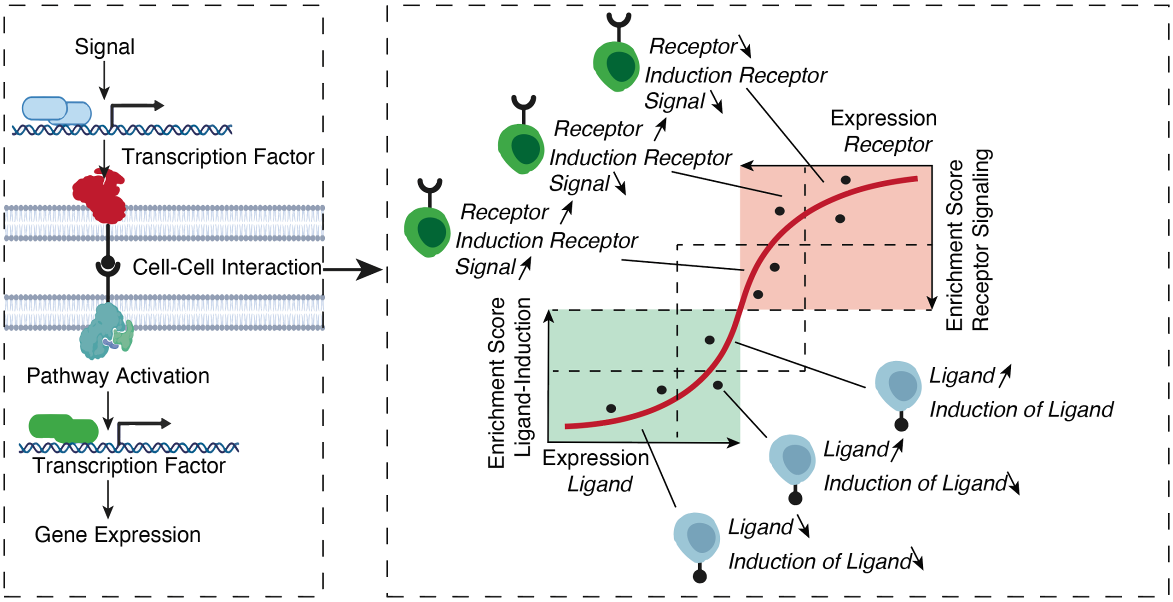

Supplementary Figure 7

1.2. NFCN Step1: Cell signaling annotation with CellChat

In the first step, we identify signaling pathways that define the up/downstream activation of any given Receptor-Ligand (R-L) interaction. The analysis is open to all R-L pairs that are present in the CellChat database. This can be accessed using: *CellChat::CellChatDB.human*

We define the following parameters from cells within the target population:

1. **Expression of the ligand gene(s):**

$$(1)\ L_{Cell^{\alpha}} = \frac{1}{n}\sum_{i=1}^{n} L_i,$$

where cells are defined as

$$Cell^{\alpha} = \{Cell^{\alpha}{}_1, Cell^{\alpha}{}_2, \dots, Cell^{\alpha}{}_{x-1}, Cell^{\alpha}\}$$

2. **The downstream activation signal:**

$$(2)\ \widehat{f_h}(S_{Cell^{\alpha}}) = \frac{1}{n}\sum_{i=1}^{n} K_h(S_{Cell^{\alpha}} - S_{Cell^{\alpha}{}_i}),$$

where K is the kernel and 0.7 > h > 0.3 is used to adjust the estimator. S defines the gene set enrichment score of the defined pathway. The gene sets are dynamically created based on the R-L input. For this dynamic adaptation, all signaling pathways that are linked to both the ligand and the receptor gene are used. The gene sets were then consolidated to identify uniquely expressed genes that take part in either downstream or upstream signaling. This de-novo assembled gene sets are then used to compute the signal activity. Enrichment scores are calculated using singular value decomposition (SVD) over individual genes within the gene set, and the coefficients of the first right-singular vector define the enrichment.

3. **Expression of the receptor gene(s):**

$$(3)\ R_{Cell^{\lambda}} = \frac{1}{n}\sum_{i=1}^{n} R_i,$$

whereby cells are defined as

$$Cell^{\lambda} = \{Cell^{\lambda}{}_1, Cell^{\lambda}{}_2, \ldots, Cell^{\lambda}{}_{x-1}, Cell^{\lambda}\}$$

4. **The downstream activation signal:**

$$(4)\ \widehat{f_h}(S_{Cell^{\lambda}}) = \frac{1}{n}\sum_{i=1}^{n} K_h(S_{Cell^{\lambda}} - S_{Cell^{\lambda}i}).$$

Similar estimation of gene set enrichment scores as illustrated in (2).

1.3. NFCN Step2: Fitting the model for cell-cell connectivity

We first integrate both source and target data after normalization (5) of both input vectors (6 and 7).

$$(5)\ n_{\mathrm{x}\,i} = \frac{A_{\mathrm{x}\,i} - min(A_x)}{max(A_x) - min(A_x)}$$

$$(6)\ EXP = L_{Cell^{\alpha}} + (2 - R_{Cell^{\lambda}}) \text{ and } (7)\ SIG = S_{Cell^{\alpha}} + (2 - S_{Cell^{\lambda}})$$

A kernel regression was then used for non-parametric estimation of the connected cells from the target and source dataset[4] (8).

$$(8)\ \widehat{\vartheta_h}(exp) = \frac{1}{n}\sum_{i=1}^{n}\int_{-\infty}^{\frac{exp-exp_i}{h}} \frac{1}{\sqrt{2\pi}} e^{-\frac{t^2}{2}dt}$$

where h is the bandwidth parameter that controls the resolution of the kernel estimation, which was optimized for each L-R pair. We then estimate the cells that are most likely to be connected by reassembling based on the connectivity coefficient (9):

$$(9)\ Source = \min_i S_{\vartheta} \longmapsto \max_i S_{\vartheta} \text{ and } Target = \min_i T_{\vartheta} \longmapsto \max_i T_{\vartheta}$$

The upper .95 quantile from both the source and target population are then defined as connected cells.

1.4. NFCN Step3: Correction of connected cells using stRNA-seq

Our model estimates the likelihood of cellular interactions between a source and target population. Without a spatial context, the likelihood of this being a physical interaction needs to be further validated. Therefore, we made use of a spatially resolved transcriptomics (stRNA-seq) dataset, to which we project both the connected and non-connected cell populations. Differentially expressed genes between connected and non-connected cells were determined and used as a basis to locate the spatial positions of the cell populations in the stRNA-seq dataset using the spotlight algorithm. Additionally, this integration of stRNA-seq datasets opens up the possibility to determine spatial

correlations between other cell types or states. The most likely position of each cell (Of the connected cell populations) was then estimated based on the top expressed genes (n=100) using

NFCN2::inferSpatialCorrection(object, inferSpatialPosition.object, assay="SCT", estimator=1, nr.genes=100).

The algorithm finally corrects the connected cell pairs based on their likely juxtaposition in space using

NFCN2::inferSpatialPosition(object, spata.obj, correction.distance = 100)).

The distance between the connected cells can be adjusted based on the type of interaction, with long distance cytokine interactions (Recommended: 100 µm) or surface marker interactions (Recommended: 50µm).

1.5. NFCN Step4: Correction based on PIC-seq

Recently, the development of PIC-seq has enabled the identification of connected cells based on physical interactions. In the presented method, doublet cell pairs were identified using fluorescence activated cell sorting (FACS) and further sequenced. Here, we use an adapted variant that makes use of the normally rejected doublets from our scRNA-seq dataset. These identified doublets can contain a variety of cell types; cells of the same type or of different cell populations. First, we identified and isolated the doublets and performed standard postprocessing by dimensional reduction and SNN clustering. The identified clusters were then separated into clusters expressing diverse and non-diverse cell type marker genes. In our case, we identified the clusters that expressed markers for both lymphoid (*CD3D*) and myeloid (*AIF1*) cells. To estimate the similarity between connected source- and target population, the top-corrected cell pairs and the lymphoid/myeloid doublet clusters were merged, followed by PCA analysis. This resulted in the doublet cluster spanning between the source and target cell population.

Bibliography

1. Efremova, M. & Vento, M. CellPhoneDB: inferring cell–cell communication from combined expression of multi-subunit ligand–receptor complexes. *Tormo*
2. Browaeys, R., Saelens, W. & Saeys, Y. NicheNet: modeling intercellular communication by linking ligands to target genes. *Nat. Methods* **17,** 159–162 (2020).
3. Jin, S. & Guerrero, C. F. Inference and analysis of cell-cell communication using CellChat. *Juarez*
4. Silverman, B. W. *Density Estimation for Statistics and Data Analysis*. (Springer US, 1986). doi:10.1007/978-1-4899-3324-9
