# Supplementary material for: T-cell dysfunction in the glioblastoma microenvironment is mediated by myeloid cells releasing interleukin-10
Source: Nat Commun. 2022 Feb 17;13:925. doi: 10.1038/s41467-022-28523-1 (PMC8854421; doi:10.1038/s41467-022-28523-1)
Supplement: Supplementary file 3 — Description of Additional Supplementary Files [file 41467_2022_28523_MOESM3_ESM.pdf]

Description of Additional Supplementary Files

File Name: Supplementary Data 1
Description: Clinical Information of all tissue donors.
