# Supplementary material for: T-cell dysfunction in the glioblastoma microenvironment is mediated by myeloid cells releasing interleukin-10
Source: Nat Commun. 2022 Feb 17;13:925. doi: 10.1038/s41467-022-28523-1 (PMC8854421; doi:10.1038/s41467-022-28523-1)
Supplement: Supplementary file 5 — Reporting Summary [file 41467_2022_28523_MOESM5_ESM.pdf]

nature research

Corresponding author(s): Dieter Henrik Heiland

Last updated by author(s): Nov 29, 2021

Reporting Summary

Nature Research wishes to improve the reproducibility of the work that we publish. This form provides structure for consistency and transparency in reporting. For further information on Nature Research policies, see our Editorial Policies and the Editorial Policy Checklist.

Statistics

For all statistical analyses, confirm that the following items are present in the figure legend, table legend, main text, or Methods section.

n/a	Confirmed	
☐	☒	The exact sample size (*n*) for each experimental group/condition, given as a discrete number and unit of measurement
☒	☐	A statement on whether measurements were taken from distinct samples or whether the same sample was measured repeatedly
☐	☒	The statistical test(s) used AND whether they are one- or two-sided<br>*Only common tests should be described solely by name; describe more complex techniques in the Methods section.*
☐	☒	A description of all covariates tested
☐	☒	A description of any assumptions or corrections, such as tests of normality and adjustment for multiple comparisons
☐	☒	A full description of the statistical parameters including central tendency (e.g. means) or other basic estimates (e.g. regression coefficient) AND variation (e.g. standard deviation) or associated estimates of uncertainty (e.g. confidence intervals)
☐	☒	For null hypothesis testing, the test statistic (e.g. *F*, *t*, *r*) with confidence intervals, effect sizes, degrees of freedom and *P* value noted<br>*Give P values as exact values whenever suitable.*
☐	☒	For Bayesian analysis, information on the choice of priors and Markov chain Monte Carlo settings
☐	☒	For hierarchical and complex designs, identification of the appropriate level for tests and full reporting of outcomes
☐	☒	Estimates of effect sizes (e.g. Cohen's *d*, Pearson's *r*), indicating how they were calculated

Our web collection on statistics for biologists contains articles on many of the points above.

Software and code

Policy information about availability of computer code

Data collection

The Single cell RNA seq data and Spatial transcriptomics data were generated using 10x Chromium controller and 10X spatial transcriptomics library preparation slide. Prepared libraries were sequenced in an llumina Nextseq 500/550 platform using High output flow cell v2.5 (150 cycles). FACS data were generated using Sony Spectral analyzer SP6800. ELISA data obtained using Tecan Infinite 200. Imaging data were generated using Observer D1: Zeiss microscope.

Data analysis

The analysis of data were achieved by using R packages. Codes used in creating the analysis is included in the methods part, where links to Github repository are also provided.
An R Session overview:
R version 4.0.1 (2020-06-06)
Platform: x86_64-apple-darwin17.0 (64-bit)
Running under: macOS Catalina 10.15.7

locale:
[1] en_US.UTF-8/en_US.UTF-8/en_US.UTF-8/C/en_US.UTF-8/en_US.UTF-8

attached base packages:
[1] stats graphics grDevices utils datasets methods base

other attached packages:
[1] forcats_0.5.1 stringr_1.4.0 dplyr_1.0.7 purrr_0.3.4 readr_2.0.1 tidyr_1.1.3 tibble_3.1.4 ggplot2_3.3.5 tidyverse_1.3.1 SPATA2_0.1.0

loaded via a namespace (and not attached):
[1] estimability_1.3 scattermore_0.7 flowWorkspace_4.0.6 coda_0.19-4 SeuratObject_4.0.2 knitr_1.33

[7] bit64_4.0.5 irlba_2.3.3 multcomp_1.4-17 DelayedArray_0.14.1 data.table_1.14.0 rpart_4.1-15
[13] RCurl_1.98-1.4 generics_0.1.0 flowCore_2.0.1 BiocGenerics_0.34.0 cowplot_1.1.1 TH.data_1.0-10
[19] RSQLite_2.2.8 RANN_2.6.1 europepmc_0.4.1 future_1.22.1 tzdb_0.1.2 bit_4.0.4
[25] enrichplot_1.8.1 spatstat.data_2.1-0 xml2_1.3.2 lubridate_1.7.10 httpuv_1.6.2
[31] assertthat_0.2.1 confuns_0.1.0 viridis_0.6.1 xfun_0.25 hms_1.1.0 promises_1.2.0.1
[37] fansi_0.5.0 progress_1.2.2 readxl_1.3.1 dbplyr_2.1.1 tmvnsim_1.0-2 Rgraphviz_2.32.0
[43] igraph_1.2.6 DBI_1.1.1 htmlwidgets_1.5.3 spatstat.geom_2.2-2 stats4_4.0.1 ellipsis_0.3.2
[49] corrplot_0.90 backports_1.2.1 ggcyto_1.16.0 cytolib_2.0.3 insight_0.14.4 RcppParallel_5.1.4
[55] deldir_0.2-10 vctrs_0.3.8 SingleCellExperiment_1.10.1 Biobase_2.48.0 ROCR_1.0-11 sjlabelled_1.1.8
[61] abind_1.4-5 batchelor_1.4.0 withr_2.4.2 cachem_1.0.6 ggforce_0.3.3 packrat_0.7.0
[67] triebeard_0.3.0 emmeans_1.6.3 sctransform_0.3.2 prettyunits_1.1.1 mnormt_2.0.2 goftest_1.2-2
[73] cluster_2.1.2 DOSE_3.14.0 lazyeval_0.2.2 crayon_1.4.1 labeling_0.4.2 pkgconfig_2.0.3
[79] tweenr_1.0.2 GenomeInfoDb_1.24.2 vipor_0.4.5 nlme_3.1-152 rlang_0.4.11 globals_0.14.0
[85] lifecycle_1.0.0 miniUI_0.1.1.1 sandwich_3.0-1 downloader_0.4 clustree_0.4.3 rsvd_1.0.5
[91] modelr_0.1.8 cellranger_1.1.0 polyclip_1.10-0 matrixStats_0.60.1 lmtest_0.9-38 graph_1.66.0
[97] datawizard_0.2.0.1 Matrix_1.3-4 urltools_1.7.3 boot_1.3-28 zoo_1.8-9 beeswarm_0.4.0
[103] reprex_2.0.1 base64enc_0.1-3 ggridges_0.5.3 GlobalOptions_0.1.2 pheatmap_1.0.12 png_0.1-7
[109] viridisLite_0.4.0 SPATA_0.1.0 parameters_0.14.0 bitops_1.0-7 ConsensusClusterPlus_1.52.0
KernSmooth_2.23-20
[115] DelayedMatrixStats_1.10.1 blob_1.2.2 shape_1.4.6 qvalue_2.20.0 parallelly_1.27.0 jpeg_0.1-9
[121] gridGraphics_0.5-1 ggeffects_1.1.1 S4Vectors_0.26.1 scales_1.1.1 memoise_2.0.0 magrittr_2.0.1
[127] plyr_1.8.6 hexbin_1.28.2 ica_1.0-2 zlibbioc_1.34.0 compiler_4.0.1 scatterpie_0.1.7
[133] RColorBrewer_1.1-2 lme4_1.1-27.1 fitdistrplus_1.1-5 cli_3.0.1 XVector_0.28.0 listenv_0.8.0
[139] patchwork_1.1.1 ncdfFlow_2.34.0 pbapply_1.4-3 FlowSOM_1.20.0 MASS_7.3-54 mgcv_1.8-36
[145] tidyselect_1.1.1 stringi_1.7.4 RProtoBufLib_2.0.0 yaml_2.2.1 GOSemSim_2.14.2 BiocSingular_1.4.0
[151] latticeExtra_0.6-29 ggrepel_0.9.1 grid_4.0.1 fastmatch_1.1-3 tools_4.0.1 future.apply_1.8.1
[157] parallel_4.0.1 rstudioapi_0.13 CytoML_2.0.5 circlize_0.4.13 gridExtra_2.3 farver_2.1.0
[163] Rtsne_0.15 ggraph_2.0.5 sjPlot_2.8.9 digest_0.6.27 rvcheck_0.1.8 BiocManager_1.30.16
[169] shiny_1.6.0 Rcpp_1.0.7 broom_0.7.9 GenomicRanges_1.40.0 performance_0.7.3 later_1.3.0
[175] RcppAnnoy_0.0.19 httr_1.4.2 AnnotationDbi_1.50.3 psych_2.1.6 effectsize_0.4.5 sjstats_0.18.1
[181] colorspace_2.0-2 rvest_1.0.1 fs_1.5.0 XML_3.99-0.7 tensor_1.5 reticulate_1.20
[187] IRanges_2.22.2 splines_4.0.1 uwot_0.1.10 yulab.utils_0.0.2 RBGL_1.64.0 spatstat.utils_2.2-0
[193] scater_1.16.2 graphlayouts_0.7.1 ggplotify_0.1.0 plotly_4.9.4.1 xtable_1.8-4 nloptr_1.2.2.2
[199] jsonlite_1.7.2 tidygraph_1.2.0 flashClust_1.01-2 ggfun_0.0.3 R6_2.5.1 pillar_1.6.2
[205] htmltools_0.5.2 mime_0.11 minqa_1.2.4 glue_1.4.2 fastmap_1.1.0 clusterProfiler_3.16.1
[211] BiocParallel_1.22.0 BiocNeighbors_1.6.0 codetools_0.2-18 fgsea_1.14.0 tsne_0.1-3 mvtnorm_1.1-2
[217] utf8_1.2.2 lattice_0.20-44 spatstat.sparse_2.0-0 ggbeeswarm_0.6.0 leiden_0.3.9 GO.db_3.11.4
[223] survival_3.2-13 munsell_0.5.0 DO.db_2.9 GenomeInfoDbData_1.2.3 haven_2.4.3 sjmisc_2.8.7
[229] reshape2_1.4.4 gtable_0.3.0 bayestestR_0.11.0 spatstat.core_2.3-0 NFCN2_0.0.0.9000 Seurat_4.0.5

For manuscripts utilizing custom algorithms or software that are central to the research but not yet described in published literature, software must be made available to editors and reviewers. We strongly encourage code deposition in a community repository (e.g. GitHub). See the Nature Research guidelines for submitting code & software for further information.

Data

Policy information about availability of data

All manuscripts must include a data availability statement. This statement should provide the following information, where applicable:

- Accession codes, unique identifiers, or web links for publicly available datasets
- A list of figures that have associated raw data
- A description of any restrictions on data availability

All the dataset that support the findings of this study are available to reviewers from the corresponding author upon request.

Field-specific reporting

Please select the one below that is the best fit for your research. If you are not sure, read the appropriate sections before making your selection.

☒ Life sciences ☐ Behavioural & social sciences ☐ Ecological, evolutionary & environmental sciences

For a reference copy of the document with all sections, see nature.com/documents/nr-reporting-summary-flat.pdf

Life sciences study design

All studies must disclose on these points even when the disclosure is negative.

Sample size	No statistical method was used to determine sample size. The sample size was chosen on the basis of prior studies that showed significant effects with similar sample sizes.
Data exclusions	Samples with RNA Integrity Number <7 were not processed. Data from contaminated samples, e.g. contaminated human organotypic cultures, were excluded. And

Replication	All experiments were reproduced to reliably support conclusions stated in the manuscript.
Randomization	Samples for Human organotypic slices cultures were randomly collected from patients undergoing glioblastoma resection. In all the cases, our results consistently proved our claims.
Blinding	The persons performing sample preparation were unaware of the project details.

Reporting for specific materials, systems and methods

We require information from authors about some types of materials, experimental systems and methods used in many studies. Here, indicate whether each material, system or method listed is relevant to your study. If you are not sure if a list item applies to your research, read the appropriate section before selecting a response.

Materials & experimental systems

n/a	Involved in the study	
☐	☒	Antibodies
☐	☒	Eukaryotic cell lines
☐	☐	Palaeontology and archaeology
☐	☐	Animals and other organisms
☐	☒	Human research participants
☐	☐	Clinical data
☐	☐	Dual use research of concern

Methods

n/a	Involved in the study	
☒	☐	ChIP-seq
☐	☒	Flow cytometry
☒	☐	MRI-based neuroimaging

Antibodies

Antibodies used	Anti- Granzyme B(mouse,ab89415,abcam), Anti-TIM3(rabbit, PA5-86067,Thermo Fisher Scientific), Anti-HMOX1(mouse, H00003162-B01P,Thermo Fisher Scientific), Anti-IBA1 (rabbit, 19-19741, FUJIFILM Wako Chemicals Europe Gmbh), anti-CD45 (rabbit, ab10558, abcam), anti-CD3(rabbit, ab5690, abcam), anti-CD3(mouse,ab8090, abcam), anti-CD68(mouse,ab955,abcam),anti-CD4(mouse,ab133616,abcam), anti-CD8(mouse,ab93278,abcam)
Validation	All antibodies were commercially available. Please see the corresponding manufacturer datasheets on webpages for reference and validation

Eukaryotic cell lines

Policy information about cell lines

Cell line source(s)	ZsGreen tagged BTSC#233 (Human)
Authentication	Cell identity was ascertained by mRNA transcriptional profiling
Mycoplasma contamination	All cell lines were tested for mycoplasma contamination. If any, it has been excluded.
Commonly misidentified lines (See ICLAC register)	*Name any commonly misidentified cell lines used in the study and provide a rationale for their use.*

Palaeontology and Archaeology

Specimen provenance	*Provide provenance information for specimens and describe permits that were obtained for the work (including the name of the issuing authority, the date of issue, and any identifying information).*
Specimen deposition	*Indicate where the specimens have been deposited to permit free access by other researchers.*
Dating methods	*If new dates are provided, describe how they were obtained (e.g. collection, storage, sample pretreatment and measurement), where they were obtained (i.e. lab name), the calibration program and the protocol for quality assurance OR state that no new dates are provided.*

☐ Tick this box to confirm that the raw and calibrated dates are available in the paper or in Supplementary Information.

Ethics oversight	*Identify the organization(s) that approved or provided guidance on the study protocol, OR state that no ethical approval or guidance was required and explain why not.*

Note that full information on the approval of the study protocol must also be provided in the manuscript.

Animals and other organisms

Policy information about studies involving animals; ARRIVE guidelines recommended for reporting animal research

Laboratory animals	*For laboratory animals, report species, strain, sex and age OR state that the study did not involve laboratory animals.*
Wild animals	*Provide details on animals observed in or captured in the field; report species, sex and age where possible. Describe how animals were caught and transported and what happened to captive animals after the study (if killed, explain why and describe method; if released, say where and when) OR state that the study did not involve wild animals.*
Field-collected samples	*For laboratory work with field-collected samples, describe all relevant parameters such as housing, maintenance, temperature, photoperiod and end-of-experiment protocol OR state that the study did not involve samples collected from the field.*
Ethics oversight	*Identify the organization(s) that approved or provided guidance on the study protocol, OR state that no ethical approval or guidance was required and explain why not.*

Note that full information on the approval of the study protocol must also be provided in the manuscript.

Human research participants

Policy information about studies involving human research participants

Population characteristics	Only patient-derived brain samples were used. Samples for sequencing experiments are from 5 males (age of 52, 59,78,65,57) and 6 females (age of 57,68,70,56,70,61).Blood samples were also collected.
Recruitment	*Describe how participants were recruited. Outline any potential self-selection bias or other biases that may be present and how these are likely to impact results.*
Ethics oversight	The local ethics committee of the University of Freiburg approved the data evaluation, imaging procedures and experimental design (protocol 100020/09 and 472/15_160880). The methods were carried out in accordance with the approved guidelines, with written informed consent obtained from all subjects. The studies were approved by an institutional review board.

Note that full information on the approval of the study protocol must also be provided in the manuscript.

Clinical data

Policy information about clinical studies

All manuscripts should comply with the ICMJE guidelines for publication of clinical research and a completed CONSORT checklist must be included with all submissions.

Clinical trial registration	*Provide the trial registration number from ClinicalTrials.gov or an equivalent agency.*
Study protocol	*Note where the full trial protocol can be accessed OR if not available, explain why.*
Data collection	*Describe the settings and locales of data collection, noting the time periods of recruitment and data collection.*
Outcomes	*Describe how you pre-defined primary and secondary outcome measures and how you assessed these measures.*

Dual use research of concern

Policy information about dual use research of concern

Hazards

Could the accidental, deliberate or reckless misuse of agents or technologies generated in the work, or the application of information presented in the manuscript, pose a threat to:

No	Yes	
☒	☐	Public health
☒	☐	National security
☒	☐	Crops and/or livestock
☒	☐	Ecosystems
☒	☐	Any other significant area

Experiments of concern

Does the work involve any of these experiments of concern:

No	Yes	
☒	☐	Demonstrate how to render a vaccine ineffective
☒	☐	Confer resistance to therapeutically useful antibiotics or antiviral agents
☒	☐	Enhance the virulence of a pathogen or render a nonpathogen virulent
☒	☐	Increase transmissibility of a pathogen
☒	☐	Alter the host range of a pathogen
☒	☐	Enable evasion of diagnostic/detection modalities
☒	☐	Enable the weaponization of a biological agent or toxin
☒	☐	Any other potentially harmful combination of experiments and agents

Flow Cytometry

Plots

Confirm that:

☒ The axis labels state the marker and fluorochrome used (e.g. CD4-FITC).

☒ The axis scales are clearly visible. Include numbers along axes only for bottom left plot of group (a 'group' is an analysis of identical markers).

☒ All plots are contour plots with outliers or pseudocolor plots.

☒ A numerical value for number of cells or percentage (with statistics) is provided.

Methodology

Sample preparation	Single-Cell suspensions were obtained after Dead-Cell Removal and CD3 MACS-enrichment. Cells were incubated with VivaFix 398/550 (BioRad Laboratories, CA, USA) according to the manufacturer´s instructions. Cells were fixed in 4% paraformaldehyde (PFA) for 10 minutes. After centrifugation (350 g; 4°C; 5 min) and removal of the supernatant, the cell pellet was suspended in 0.5 ml 4°C cold FACS buffer. Cell suspensions were washed and centrifuged at 350xg for 5 mins, followed by resuspension in FACS buffer. The washing step was repeated twice. Finally, cells were resuspended in at least 0.5 to 1 mL of FACS buffer depending on the number of cells. We used a Sony SP6800 spectral analyzer in standardization mode with PMT voltage set to maximum to reach a saturation rate below 0.1 %. Gating was performed
Instrument	Sony Spectral analyzer SP6800
Software	SP6800
Cell population abundance	Purity was determined by running a purity check of the sorted populations after the sort was completed.
Gating strategy	The gating strategy is mentioned in supplementary figure 1.

☒ Tick this box to confirm that a figure exemplifying the gating strategy is provided in the Supplementary Information.
